# Dairy Products: Processing Technology and Sensory Properties

**DOI:** 10.3390/foods13162551

**Published:** 2024-08-16

**Authors:** Michele Faccia, Giuseppe Natrella

**Affiliations:** Department of Soil, Plant and Food Sciences, University of Bari, Via Amendola 165/A, 70126 Bari, Italy; disspa@pec.uniba.it

In developed countries, the dairy sector is going through a highly challenging phase as a consequence of changes in consumers’ expectations and the spread of new cultural approaches to food. Among the most challenging requirements are producing products with a more balanced chemical composition, valorizing or improving their nutraceutical properties, extending shelf life to reduce food waste, and finding new tools to enhance traceability and safety. Of course, all these goals must be reached without impairing foods’ sensory characteristics, which remain a constraint in food choices. This Special Issue comprises several interesting contributions to the field authored by researchers from 11 different countries. The contents of these papers can be grouped into three categories: the development and consumer acceptance of innovative products with improved nutritional characteristics, the effect of processing on quality and traceability, cheese shelf life, and preservation conditions.

In Article 1, an interesting functional dairy product was developed consisting of a buttermilk-based fermented drink fortified with blueberry pomace, a fruit byproduct. This is an innovation creation given that the literature related to this research largely focuses on the fortification of buttermilk with industrial or self-produced ingredients but not with byproducts [1,2,3]. The use of bovine colostrum in cheesemaking was studied in Article 2, with the aim of developing fresh and matured cheeses rich in bioactive compounds. Colostrum differs significantly from milk and contains higher concentrations of bioactive compounds, such as immunoglobulins, enzymes, vitamins, and growth factors, but its presence in milk may cause problems in industrial processes [4]. After chemical, microbiological, and sensory evaluation, the authors found the presence of colostrum is compatible with the production of fresh cheese at a 75:25 ratio. The authors of Article 3 produced Pecorino Cheese from the milk of sheep whose diet had been supplemented with flaxseed and algae (*Ascophyllum nodosum*) and registered a higher content of unsaturated fatty acids and lower levels of atherogenic and thrombogenic indexes than in the control cheese. This improvement in nutritional quality did not have a detrimental impact on the cheese’s sensory attributes.

Four research groups contributed to this Special Issue by publishing papers dealing with the effect of processing on the quality and traceability of dairy products. In Article 4, the possibility of using concentrated demineralized liquid whey in the formulation of ice cream was investigated. This study also considered two processing variables: the addition of lactase to the whey concentrate to hydrolyze lactose and fortification with whey protein isolate. The obtained products were subjected to a series of laboratory analyses, with a particular focus on the physical characteristics. The results were thoroughly discussed, and the effect of all the variables was evaluated by highlighting the pros and cons of the different formulations. An interesting study was conducted on the effect of processing at the level of microRNAs in milk and milk-related products (Article 5). MiRNAs are noncoding RNAs that are present in milk and might have bioactive effects in humans. Research on these molecules as possible biomarkers of the dairy system, diet, and animal health status is rapidly increasing worldwide [5,6,7], but more information is needed about the effect of milk processing on their presence. In this study, the levels of seven microRNAs were assessed in raw milk and three derived products: microwaved milk, yogurt, or cheese. The results demonstrated that milk treatments tended to decrease the level of all the miRNAs to some extent, even though the concentration effect that took place during cheesemaking counterbalanced this decrease. In conclusion, raw milk and cheese may contain similar concentrations of miRNAs, which are higher than those of yogurt and microwaved milk. 

The effect of applying milk thermization in the production of Canestrato Pugliese, a Protected Designation of Origin (P.D.O.) ovine hard cheese, was assessed in Article 6. According to the official production protocol, this cheese can only be manufactured from raw milk, with a high risk of defects that might undermine the profitability of the cheesemaking process [8,9]. The results obtained in this study demonstrated that heat treatment did not lead to remarkable differences in the gross composition with respect to cheese made from raw milk, but caused different microbiological profiles that led to differences in proteolysis during ripening. The sensory analysis revealed that the thermized cheese lost some of its typical sensory characteristics, which was likely due to a reduction in indigenous microbiota populations. It was concluded that milk thermization could only be applied with the development and use of an autochthonous starter. 

Article 7 focused on the application of vacuum-assisted block freeze concentration (BFC) to concentrate different types of whey. In BFC, the liquid is completely frozen, and the temperature at the core of the product is set to below its freezing point. Subsequently, the block is thawed, and the concentrated fraction is then separated from the ice fraction [10,11]. During the process, a vacuum pressure can be applied. The study first investigated the influence of the initial concentration, time, and vacuum pressure on the concentration index and solute yield; then, the optimal time and vacuum pressure conditions were applied to three different types of whey. Additionally, the effect of vacuum-assisted BFC on lactose content was also studied, and the results suggested that lactose tends to remain in the concentrated phase rather than in the ice. In this way, it is possible to recover, in a single step, at least 70% of the lactose initially contained in the whey. 

Finally, three papers focused on the shelf life of cheese and its preservation conditions. The effect of two different types of preservation containers (stainless steel tanks—SST, and tin containers—TC) on the cheeses’ physicochemical, microbiological, and textural characteristics was investigated in Article 8, along with the volatile profile of white brined cheese. The results of this study showed that the material and capacity of the ripening–preservation containers did not statistically significantly affect the physicochemical, textural, microbiological, and sensory characteristics of the white brine cheeses. The authors concluded that stainless steel tanks can be used by cheese factories with a significant focus on repackaging, as an SST container keeps dairy products fresher at lower temperatures, as well as having the advantages of being reusable and highly resistant to corrosion.

Article 9 focused on the shelf-life extension of Burrata cheese, a fresh pasta filata cheese that is becoming very popular worldwide. This cheese is very similar to mozzarella and is increasingly manufactured at an industrial level. The same strategies proposed for extending the shelf life of mozzarella [12,13,14] have also been tested on burrata, but with poor results. In this study, the combination of a commercial bioprotective starter and modified-atmosphere packaging (MAP) was evaluated as a strategy to delay the spoilage of a product’s quality. The main outcome of the research was that a synergy between the modified atmosphere and bioprotective starter, in conjunction with good-quality raw materials and good manufacturing practices, can significantly improve the microbiological stability of burrata without the use of chemical additives. Finally, Albisu et al. (Article 10) evaluated the influence of different types of packaging conditions—air, vacuum, and four different modified atmospheres—on the quality of semihard Idiazabal sheep’s milk cheese ripened for 56 days under refrigerated conditions. MAP was found to be the most effective preservation technique when compared to air- and vacuum-packaging treatments. Air-packaged cheeses presented a moldy flavor by day 35, whereas vacuum packaging led to a paste-like appearance and holes after 14 days. MAP mixtures with a CO_2_ concentration between 50/50 and 80/20% CO_2_/N_2_ (*v*/*v*) were found to ensure sensory quality and stability in the distribution of these raw sheep-milk cheese wedges.

In summary, the Special Issue “Dairy Products: Processing Technology and Sensory Properties” has supplied some new information to help combat modern challenges faced by the dairy sector and has demonstrated that innovative research in this sector is very active.
